# Dietary Selenium Supplementation Ameliorates Female Reproductive Efficiency in Aging Mice

**DOI:** 10.3390/antiox8120634

**Published:** 2019-12-11

**Authors:** Haoxuan Yang, Izhar Hyder Qazi, Bo Pan, Christiana Angel, Shichao Guo, Jingyu Yang, Yan Zhang, Zhang Ming, Changjun Zeng, Qingyong Meng, Hongbing Han, Guangbin Zhou

**Affiliations:** 1 Farm Animal Genetic Resources Exploration and Innovation Key Laboratory of Sichuan Province, College of Animal Science and Technology, Sichuan Agricultural University, Chengdu 611130, China; yanghaoxuan940712@gmail.com (H.Y.); vetdr_izhar@yahoo.com (I.H.Q.); bopan1992@163.com (B.P.); daxiaopang@outlook.com (S.G.); yangjinyu19960710@163.com (J.Y.); yanzhang@sicau.edu.cn (Y.Z.); zhangming@sicau.edu.cn (Z.M.); zengchj@sicau.edu.cn (C.Z.); 2 Department of Veterinary Anatomy and Histology, Shaheed Benazir Bhutto University of Veterinary and Animal Sciences, Sakrand 67210, Pakistan; 3 Department of Veterinary Parasitology, College of Veterinary Medicine, Sichuan Agricultural University, Chengdu 611130, China; qazi5502@yahoo.com; 4 Department of Veterinary Parasitology, Faculty of Veterinary Sciences, Shaheed Benazir Bhutto University of Veterinary and Animal Sciences, Sakrand 67210, Pakistan; 5 State Key Laboratory of AgroBiotechnology, China Agricultural University, Beijing 100193, China; qymeng@cau.edu.cn; 6 National Engineering Laboratory for Animal Breeding, Key Laboratory of Animal Genetics and Breeding of the Ministry of Agriculture, Beijing Key Laboratory for Animal Genetic Improvement, College of Animal Science and Technology, China Agricultural University, Beijing 100193, China

**Keywords:** selenium, ovarian aging, *Gpx1*, *Gpx3*, GPX4, *Selenof*, selenoprotein, follicle development, embryo, apoptosis

## Abstract

Female reproductive (ovarian) aging is distinctively characterized by a markedly reduced reproductive function due to a remarkable decline in quality and quantity of follicles and oocytes. Selenium (Se) has been implicated in playing many important biological roles in male fertility and reproduction; however, its potential roles in female reproduction, particularly in aging subjects, remain poorly elucidated. Therefore, in the current study we used a murine model of female reproductive aging and elucidated how different Se-levels might affect the reproductive efficiency in aging females. Our results showed that at the end of an 8-week dietary trial, whole-blood Se concentration and blood total antioxidant capacity (TAOC) were significantly reduced in Se-deficient (0.08 mg Se/kg; Se-D) mice, whereas both of these biomarkers were significantly higher in inorganic (0.33 mg/kg; ISe-S) and organic (0.33 mg/kg; OSe-S) Se-supplemented groups. Similarly, compared to the Se-D group, Se supplementation significantly ameliorated the maintenance of follicles and reduced the rate of apoptosis in ovaries. Meanwhile, the rate of in vitro-produced embryos resulting from germinal vesicle (GV) oocytes was also significantly improved in Se-supplemented (ISe-S and OSe-S) groups compared to the Se-D mice, in which none of the embryos developed to the hatched blastocyst stage. RT-qPCR results revealed that mRNA expression of *Gpx1*, *Gpx3*, *Gpx4, Selenof*, *p21*, and *Bcl-2* genes in ovaries of aging mice was differentially modulated by dietary Se levels. A considerably higher mRNA expression of *Gpx1*, *Gpx3*, *Gpx4*, and *Selenof* was observed in Se-supplemented groups compared to the Se-D group. Similarly, mRNA expression of *Bcl-2* and *p21* was significantly lower in Se-supplemented groups. Immunohistochemical assay also revealed a significantly higher expression of GPX4 in Se-supplemented mice. Our results reasonably indicate that Se deficiency (or marginal levels) can negatively impact the fertility and reproduction in females, particularly those of an advancing age, and that the Se supplementation (inorganic and organic) can substantiate ovarian function and overall reproductive efficiency in aging females.

## 1. Introduction

Aging is a slow and complex natural process leading to a gradual, progressive, and irreversible decline in several physiological functions of the body [1]. However, in contrast to aging in other major organs and systems, female reproductive (ovarian) aging, as a unique biological phenomenon, usually occurs midway through the lifespan [2,3], and is distinctively characterized by a markedly reduced reproductive function due to a considerable decline in quality and quantity of follicles and oocytes in the ovary [4,5]. As a paradigm of mammalian reproduction, it is generally well-known that females are usually born with a fixed oocyte quantity (a true reserve of primordial follicles) which continuously declines until a few follicles or oocytes remain, leading to ovarian aging [5]. In addition, cyclic folliculogenesis and ovulation, as well as massive follicular atresia via apoptosis, lead to the ovarian atrophy and also contribute to female reproductive aging [4]. Similarly, the age-associated poor quality of follicles/oocytes is linked to chromosomal abnormalities (misalignment and aneuploidy), meiotic spindle anomalies, telomere shortening, mitochondrial dysfunction, apoptosis, DNA mutations, and protein damage, all of which collectively contribute to an accelerated ovarian aging and increased risk of infertility [2,4]. It has been reported that low-quality follicles/oocytes have considerably reduced competence for further development [6]. Although our understanding of the role of oxidative stress in mammalian ovarian aging is still incomplete, it has been shown that increased levels of reactive oxygen species (ROS) and accumulating oxidative stress during ovarian aging contribute to follicular atresia and the resultant quantitative and qualitative decline in oocyte reserves [7]. Similarly, an increased ROS generation is also shown to negatively impact several critical physiological processes related to oocyte maturation, fertilization, and embryo development [7]. Previously it was shown that increased levels of ROS, oxidative stress, and impaired antioxidant status and redox homeostasis led to an increased rate of apoptosis in ovarian follicles and granulosa cells in aging mice [4,6,7]. During last few years, antioxidants such as N-acetyl-l-cysteine (NAC) [6], resveratrol [4], and melatonin (MT) [8] have been demonstrated to ameliorate the ovarian redox status and oocyte and embryo quality, thereby delaying the age-related decline in fertility in mouse models of reproductive aging.

Selenium (Se) is known as an essential trace mineral (micronutrient) and performs several important functions at the level of the cell and the organism in animal and human health [9,10,11]. Although the cellular biochemistry of Se and its regulation in body are complex processes [12,13,14], it is well-established that the canonical biological roles of Se are mainly performed by selenoproteins which are encoded by 25 genes in humans and 24 in mice [9,15]. Se, as an essential component, is incorporated into selenoproteins that have selenocysteine (Sec), the twenty-first “naturally occurring” proteinogenic amino acid in the genetic code, residues at their primary “active center” [12,14,15]. Sec is encoded by an in-frame UGA codon, whose usual role is to terminate the translation [16]. Although, not all the selenoproteins have been functionally characterized, the majority of them perform oxidoreductase functions [15]. Due to their diverse properties, selenoproteins have been demonstrated to serve a number of physiological processes, including male [10] and female [9] reproduction.

Selenium is generally utilized in the form organic and inorganic species [10,17,18,19]. Selenomethionine (SeMet) is the most common organic form (mainly Se-yeast) of Se present in a wide range of human and animal diets, whereas inorganic chemical forms such as, sodium selenite are usually used for supplementing animal feed [18,19,20]. It has been argued that the potential beneficial and/or adverse effects of Se are mainly dependent on the form being utilized; however, the total intake of Se has also been considered as an important factor in this regard [10,19]. The bioavailability of organic Se species such as, SeMet is usually higher compared to the inorganic selenite and selenate species [13,17]. It should be noted that regardless of the chemical form of Se, selenide (H_2_Se) is believed to be a central point in the complex series of metabolic inter-conversions of both organic and inorganic Se species [13]. The response curve of Se is U-shaped with a constringe window between deficient and excessive intakes [11]. Both Se deficiency and excess levels have been shown to influence the redox status and lead to severe adverse health consequences in animals and humans [10,21]. Interestingly, recently it has been demonstrated that plasma Se concentrations tend to decline in certain healthy human populations comprised of older subjects [22]. It was also reported that Se bioavailability was impacted by aging [13,23].

It has been proposed that dietary Se deficiency is potentially linked to a tradeoff between health decline and longevity [24]. For instance, recently it was demonstrated that long-term deficiency of dietary Se was linked to accelerated aging and aging-related phenotypes in mice [25]. Recent evidence suggests that Se and selenoproteins are plausibly implicated in impacting the process of aging, particularly through regulation of redox homeostasis, redox signaling pathways, and genome maintenance [1,24,26,27]. This caveat is supported by the notion that the process of aging could be modulated by a sub-group of low-hierarchy selenoproteins whose expression levels are preferentially decreased in Se-deficient conditions. However, it is worth mentioning that, although selenoproteins are implicated in Se regulation in aging and age-associated functional decline, our understanding of precise mechanistic and functional basis of such regulation at the level of individual selenoproteins is still incomplete [1,24].

Dietary deficiency of essential trace elements such as Se has been shown to impair the mammalian reproductive physiology [9,10,28]. In a well-known previous study on 112 patients undergoing in vitro fertilization (IVF) procedures, Paszkowski and colleagues [29] reported that detectable traces of Se were observed in human follicular fluid, and it was shown that subjects with idiopathic infertility had significantly lower Se levels compared to those who were suffering from known causes of infertility. It was also suggested that glutathione peroxidase (GPX) could play an essential role in the follicular microenvironment and thus promote gametogenesis and fertilization [29]. However, despite past evidence of Se implication in female fertility and reproduction, there are more studies focusing on the role of Se in male reproduction [10]. There is still a comparative dearth of adequately powered mechanistic and functional studies elucidating the wider implications of Se and selenoproteins in female fertility and reproduction [9,28]. Available evidence highlights that studies focusing on role of Se as an essential trace element in female reproduction are mainly focused on human pregnancy [30] and related complications such as pre-eclampsia [31], miscarriage and preterm birth [9,32], intrauterine growth restriction [33], small-for-gestational age newborns [34], pregnancy-induced hypertension [35], and pregnancy-related autoimmune thyroid disease [36]. Lower Se status and glutathione peroxidase 3 (GPX3) activity are shown to contribute in the progression of neoplastic diseases of female reproductive system including endometrial and ovarian cancers [37,38,39,40]. In addition, a few recent reports have also demonstrated that Se deficiency was linked to the contractile dysfunction of uterine smooth muscles in young mice [41,42,43]. It was shown that Se was implicated in regulating the physiology of uterine smooth muscles potentially via modulating the expression of *Selenon*, *Selenot*, and *Selenow* [41,42], and the G protein Rho (RhoA)/Rho kinase (ROCK) signaling pathway in mice [43]. However, only recently have researchers paid attention to potential role of Se in ovarian physiology and embryo development [9,28,44,45,46,47,48,49,50]. Interestingly, when exploring the mainstream literature, with the exclusion of one study reporting the beneficial effects of Se supplementation on sperm parameters in aging mice [51], no study has yet elucidated how Se might ameliorate the reproductive function in aging mammalian models, let alone the aged females. Therefore, in this study, using a mouse model of female reproductive aging, we demonstrate that dietary Se supplementation (inorganic and organic forms) can ameliorate the Se deficiency- and age-related decline in ovarian function and reproductive efficiency in aged females.

## 2. Materials and Methods

### 2.1. Ethics Statement

All experimental protocols performed during this research were carried out in complete accordance to the regulatory guidelines of Animal Ethical and Welfare Committee (AEWC) of Sichuan Agricultural University, China (approval code: AEWC2016, 6 January 2016).

### 2.2. Animals and Experimental Groups and Diet Regimes

In this study, a total of 90 female ICR mice (Dashuo company, Chengdu, China) were used as murine model of reproductive aging (age = 12 months). Mice were provided with a standard housing environment [52]. Following a two-week adaptation period, mice were randomly divided (*n* = 18 each) into five groups i.e., Group 1, Group 2, Group 3, Group 4, and Group 5. At the start of an 8-week feeding trail, mice in all groups were fed a Se-deficient (Se-D) diet (sodium selenite 0.08 mg Se/kg) for initial two weeks to adapt to the experimental diets and to deplete their Se stores to equate the baseline blood Se status [53]. For the next six weeks, mice in different groups (Groups 1–5) were retagged as Se-deficient (Se-D), inorganic Se-adequate (ISe-A), inorganic Se-supplemented (ISe-S), organic Se-adequate (OSe-A), and organic Se-supplemented (OSe-S) groups, respectively. They received one of the following diets with different concentrations of inorganic and organic Se—Se-D: sodium selenite 0.08 mg Se/kg; ISe-A: sodium selenite 0.15 mg/kg; ISe-S: sodium selenite 0.33 mg/kg; OSe-A: Se-yeast 0.15 mg/kg; and OSe-S: Se-yeast 0.33 mg/kg.

### 2.3. Blood Analyses

For analysis of whole-blood Se concentration and blood plasma total antioxidant capacity (hereinafter called blood TAOC), the blood samples were collected at weeks 2 (baseline value at start of the feeding trial) and 8 (endpoint). The blood samples collected for determining whole-blood Se concentration were immediately processed. As for blood TAOC, plasma samples were isolated and stored at −80 °C until analyzed.

#### 2.3.1. Determination of Whole-Blood Se Concentrations

Whole-blood Se concentrations were determined by hydride generation atomic fluorescence spectrometry (HG-AFS), which is a well-established analytical method of Se determination in biological samples. Briefly, blood samples were acid-digested (HNO_3_-HClO_4_; 9:1) and reduced (hexavalent Se (VI) to tetravalent Se (IV)) in 6 mol/L hydrochloric acid (HCL) medium. Then, Se was further reduced to hydrogen selenide using sodium tetra hydroborate as a reducing agent. The reduced Se form i.e., hydrogen selenide, was carried into the atomizer by a carrier gas (argon gas) for atomization. The fluorescence of the characteristic wavelength, whose fluorescence intensity is proportional to the Se content, was quantified and compared to the standard series. All samples and standards were run in duplicates. Instrument (Atomic fluorescence spectrometer) performance conditions were as follows: negative high voltage 340 V; lamp current 100 mA; atomization temperature 800 °C; furnace height 8 mm; carrier gas flow rate 500 mL/min; shielding gas flow rate 1000 mL/min.

#### 2.3.2. Determination of Blood Total Antioxidant Capacity

Blood total antioxidant capacity (TAOC) was assayed using a commercial kit (Nanjing Jiancheng Bioengineering Research Institute, Nanjing, Jiangsu, China) according to the manufacturer’s instructions (A015-3 FRAP method).

### 2.4. Ovarian Tissue Specimen Collection

At the end of experimental feeding trial i.e., at week 8, some of the mice in all experimental groups were randomly collected and humanly euthanized. Ovaries were collected immediately for subsequent histological and immunohistochemical (IHC) analyses.

#### 2.4.1. Histological Assessment for Follicle Counting

Freshly collected ovarian tissues were fixed in 4% paraformaldehyde (8–24 h), dehydrated in a graded series of ethanol, cleared in xylene and finally embedded in paraffin wax. Paraffin-embedded specimens were serially sectioned (5 µm) using a rotary microtome (Leica RM2135, Germany). Sections were mounted on clean glass slides and then stained with routine hematoxylin and eosin (H and E) stain. For quantitative assessment of primordial and primary follicles, sections were analyzed using a robust unbiased stereological method as described previously [54]. Briefly, for follicle counting, every 10th and 11th section was sampled with a random start in the first five sections, where the 10th section served as a “reference section” and the other the “look up” section. Raw follicle counts were obtained and substituted in reciprocal fractions to get the total estimated number of primordial and primary follicles per ovary. For secondary and antral follicles and corpora lutea, exact counts were obtained by using every fifth section encompassing whole cross-section of the ovary. Based on histomorphological features, follicles were identified and categorized as follows: primordial follicles: follicles with a single partial or complete layer of squamous granulosa cells; primary follicles: with a complete single layer of cuboidal granulosa cells; secondary follicles: with two complete layers of cuboidal granulosa cells and no visible antrum; and antral follicles: follicles exhibiting formation of a fluid-filled cavity i.e., antrum adjacent to the oocyte [54]. All histological observations were made using a light microscope (Olympus BX53, Tokyo, Japan). Representative photomicrographs (TIFF format) were obtained using a built-in digital camera (Olympus-DP80, Tokyo, Japan).

#### 2.4.2. Immunohistochemistry for GPX4 Expression Analysis

For IHC assay, formalin-fixed paraffin-embedded (FFPE) tissue sections were de-paraffinized and rehydrated in a graded series of ethanol. Then, the specimens were washed in tap water to remove excessive ethanol traces. Next, specimen slides were placed in a microwaveable container and subjected to heat-induced epitope retrieval (HIER) step using a lab-grade microwave oven. This step was completed by placing the slides in an EDTA antigen retrieval buffer (pH 9.0; Cat. No. G1201; Servicebio Technology Co. Ltd. Wuhan China). Slides were allowed to cool (around 15 min) and placed in phosphate-buffered saline (PBS; pH 7.4) and washed three times (5 min each). Endogenous peroxidase activity was blocked by incubating the specimens with 3% aqueous solution of hydrogen peroxide (H_2_O_2_) for 25 min in the dark at room temperature. Slides were then placed in PBS (pH 7.4) and washed three times (5 min each). Serum blocking was performed by incubating the specimens with 3% bovine serum albumin (BSA) and rabbit serum for 30 min at room temperature. Then the specimens were incubated with the primary antibody (Rabbit monoclonal anti-Gpx4; Cat. No. Ab125066; Abcam, Shanghai, China) diluted (1:100) in PBS at 4 °C overnight in a humidified box. In the next step, slides were placed in PBS (pH 7.4) and washed three times (5 min each). Then, specimen slides were incubated with secondary antibody (ready to use HRP-labeled goat anti-rabbit; Cat. No. G1211; Servicebio Technology Co. Ltd. Wuhan, China). Then, the slides were washed again three times (5 min each) and freshly prepared DAB coloring solution (DAB chromogenic substrate kit, Cat. No. G1211; Servicebio Technology Co. Ltd. Wuhan, China) was added dropwise. The color development time was controlled under a microscope. After development of positive color (brownish yellow), tap water was applied to terminate further color development. Finally, slides were counterstained with hematoxylin for 3 min and rinsed with tap water. Hematoxylin differentiation solution (Cat. No. G1039; Servicebio Technology Co. Ltd. Wuhan, China) was applied for a few seconds and slides were again rinsed with tap water, dehydrated, cleared, slightly dried, and sealed with a neutral gum. Slides were examined using a research quality microscope (Olympus BX53, Tokyo, Japan) and photomicrographs (TIFF format) acquisition was done using a built-in high definition digital camera (Olympus-DP80, Tokyo, Japan). The positive areas of IHC staining (brownish yellow) were analyzed using Image Pro Plus software (v. 6.0.0.260; Media Cybernetics, Rockville, MD USA). Average optical density (AOD) was used to assess the expression/signal intensity of GPX4 in the ovarian tissues.

#### 2.4.3. Detection of Apoptosis in Ovarian Tissues by TUNEL Assay

The terminal dexynucleotidyl transferase (TdT)-mediated dUTP nick end labeling (TUNEL) assay was performed using an apoptosis detection kit according to the manufacturer’s instructions (Roche Diagnostics, Basel, Switzerland). Briefly, FFPE ovarian tissue sections were cut at a thickness of 5 μm and mounted on clean glass slides. Specimens were incubated with proteinase K for 30 min in a 37 °C incubator. Slides were placed in PBS (pH 7.4) and washed three times (5 min each). Next, for blocking the endogenous peroxidase activity, specimens were incubated with 3% H_2_O_2_ for 15 min in the dark at room temperature. Slides were placed in PBS (pH 7.4) and washed three times (5 min each). Then, reagent 1 (TdT) and reagent 2 (dUTP) (from TUNEL kit) were mixed (2:29) and specimens were incubated for 2 h at 37 °C in a humidified chamber. Specimens were again placed in PBS (pH 7.4) and washed three times (5 min each). Specimens were incubated (in a wet box) with Reagent 3 (converter-POD) at 37 °C for 30 min. After washing (PBS (pH 7.4) 3 × 5 min each), diaminobenzidine (DAB) coloring solution was applied for 10–15 min to stain the nuclei of apoptotic cells. The positive nuclei were stained brownish yellow. Harris hematoxylin was used for counterstaining. Prepared specimens were dehydrated in a graded series of ethanol and incubated in xylene for clearing. Finally, slides were dried and mounted with neutral gum seal. Slides were examined under a microscope (Olympus BX53, Tokyo, Japan) and the ratio of apoptotic cells (TUNEL-positive) to total cells counted was quantified using Image Pro Plus software (version: 6.0.0.260; Media Cybernetics, Rockville, MD USA). Representative fields were photographed using a high-definition built-in digital camera (Olympus-DP80, Tokyo, Japan). These observations were made in ten randomly selected microscopic fields and the numbers were then averaged.

### 2.5. Total RNA Extraction and cDNA Synthesis

Total RNA extraction was performed using Trizol reagent (15596-026; Ambion company, Austin, TX, USA) according to the manufacturer’s guidelines. Then, the total extracted RNA was treated with the gDNA Eraser Kit (5× gDNA Eraser Buffer: 2 μL, gDNA Eraser: 1 μL, total RNA: 2 μL, and, RNase Free H_2_O: 5 μL) to remove the genomic DNA. The quality and concentration of RNA were evaluated using a spectrophotometer (NanoDrop ND1000; NanoDrop Technologies, Hampton, NH, USA). Complementary DNA (cDNA) was synthesized from 1 μg total RNA using Takara Bios RR047A Synthesis kit (Takara, Kusatsu, Shiga Prefecture, Japan) according to the manufacturer’s guidelines.

### 2.6. Quantitative Reverse Transcription PCR (RT-qPCR)

For relative mRNA expression analysis, we performed real-time RT-qPCR assay using CFX 96TM Real-Time PCR Detection System (Bio-Rad, Hercules, CA, USA). Real-time RT-qPCR assay was performed using the Takara RR820A kit (Takara, Japan) as per the manual instructions and our laboratory practice. For this purpose, we used a 10-μL reaction mixture containing 1 μL cDNA, 5 μL TB Green Premix Ex Taq II (Takara, Kusatsu, Shiga Prefecture, Japan), 0.5 μL each of the forward and reverse primers, and 3 μL ddH_2_O. Glyceraldehyde 3-phosphate dehydrogenase (*Gapdh*) was used as an internal reference gene for normalization. The PCR reaction conditions were as follows: (1) an initial denaturation at 95 °C for 3 min; (2) 40 cycles of 95 °C for 10 s; and (3) primer-specific annealing temperature for 30 s (Table S1). A melting curve analysis was performed to validate the reaction specificity. Only one product of desired size was identified, and a single smooth peak was observed for each primer in melt curve analyses. Each sample was analyzed in triplicate. Primer pairs for all genes were designed using Primer Premier 5.0 software (PREMIER Biosoft International, Paulo Alto, CA, USA), except for *Gapdh*, for which we used a previously designed primer pair as reported in our recent publication [52]. The details of primers used in this study are shown in Table S1. The relative mRNA expression levels were calculated using 2^−ΔΔCt^ (Livak) method [55].

### 2.7. Superovulation, GV Oocyte Collection and In Vitro Culture

#### 2.7.1. Oocyte Collection and In Vitro Maturation (IVM)

At the end of feeding trial i.e., at week 8, some of the mice from all groups were randomly selected and subjected to superovulation. Briefly, mice were induced to superovulate by an intraperitoneal injection of 10 IU equine chorionic gonadotropin (PMSG, NingBo second hormone factory, Ningbo, China). Then, 44–46 h post-PMSG injection, mice in all groups were humanly euthanized (cervical dislocation) and ovaries were collected immediately for subsequent collection of germinal vesicle (GV) oocytes. Using a stereomicroscope with a built-in warming (37.5 °C) stage, GV oocytes were collected immediately by follicle puncturing with a syringe needle, and placed in M2 medium. The selected GV oocytes were placed in M16 medium and cultured in vitro in an incubator (Thermo Electron Corporation, Marietta, OH, USA) at 37.5 °C and 5% CO_2_. After 14 h in vitro culture, matured oocytes (MII) were selected under a stereomicroscope (with a built-in warming stage) for further experiments.

#### 2.7.2. Oocytes Activation and Embryonic Development

GV oocytes in vitro matured to MII oocytes (group-wise) were parthenogenetically activated. Briefly, MII oocytes in each group were transferred to a parthenogenetic activation (PA) medium (Ca^2+^-free human tubal fluid (HTF) supplemented with 10 mmol/L SrCl_2_ and 2 μg/mL cytochalasin D). After washing thrice in an activation medium, oocytes were incubated first in the activation medium for 2.5 h and then in a regular HTF supplemented with 2 μg/mL cytochalasin D and incubated for 3.5 h at 37.5 °C in a 5% CO_2_ incubator. For embryo culture, oocytes were rinsed three times and cultured in KSOM-AA medium. After 24, 96, and 120 h in vitro culture, two-cell embryos, blastocysts, and hatching blastocysts were observed and counted, respectively to assess developmental competence.

### 2.8. Statistical Analyses

As appropriate, statistical differences in the data were analyzed using either a Student t test or one-way analysis of variance (ANOVA) followed by a Duncan’s post-hoc test. All statistical analyses were performed using SPSS statistical software (v. 20.0, IBM, Chicago, IL, USA). Data are expressed as mean ± SEMs. A *p* value of ≤ 0.05 was considered statistically significant.

## 3. Results

### 3.1. Baseline Whole-Blood Se Status after Initial Two-Week Feeding with a Low-Se Diet

**Figure 1:**
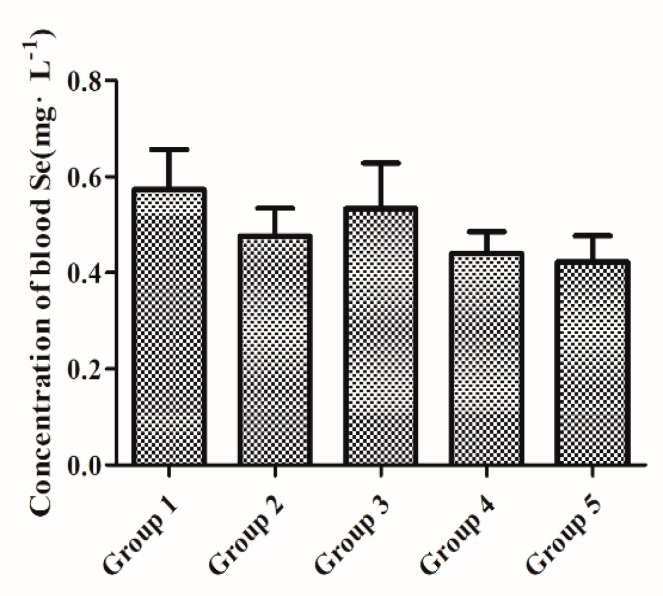
Baseline (week 2) whole-blood selenium (Se) levels in aging female mice fed a Se-deficient (sodium selenite 0.08 mg/kg) diet for initial two weeks. Each group comprised of three independent replicates (*n* = 6 each). Values are expressed as mean ± SEMs. Note: In subsequent experiments and assays, Groups 1, 2, 3, 4, and 5 were tagged as Se-D, ISe-A, ISe-S, OSe-A, and OSe-S, respectively, and received the corresponding concentrations of dietary Se as mentioned in Section 2.2.

Initial two-week feeding with a Se-D diet (0.08 mg/kg Se) was sufficient to stabilize the whole-blood Se concentration between all groups. As depicted in Figure 1, no significant differences (*p* > 0.05) were observed in whole-blood Se concentration between all experimental groups at baseline (week 2).

### 3.2. Endpoint Whole-Blood Se Status in Aging Mice Fed Different Concentrations of Se

**Figure 2:**
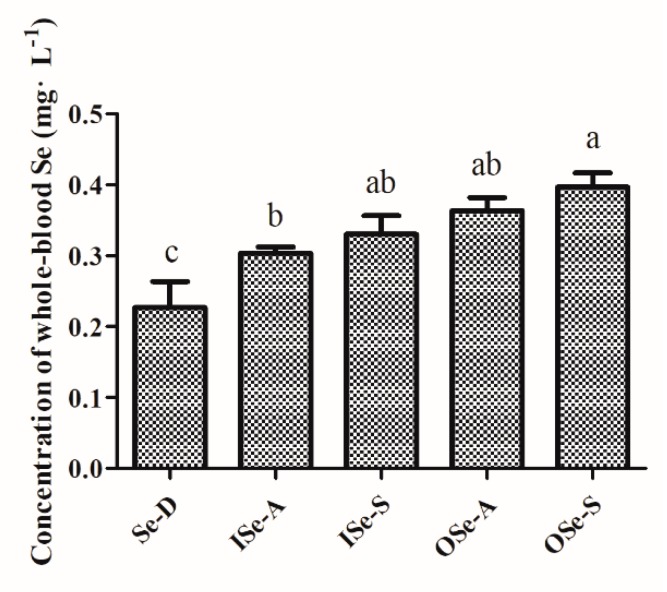
Endpoint (week 8) whole-blood Se levels in aging female mice fed different concentrations of selenium (Se) for six weeks. Values are expressed as mean ± SEMs. Each group comprised of three independent replicates (*n* = 6 each). Different letters (a, b, and c) indicate significant difference (*p* < 0.05). Notes: Se-D: Se-deficient (sodium selenite 0.08 mg/kg); ISe-A: inorganic Se-adequate (sodium selenite 0.15 mg/kg); ISe-S: inorganic Se-supplemented (sodium selenite 0.33 mg/kg); OSe-A: organic Se-adequate (Se-yeast 0.15 mg/kg); OSe-S: organic Se-supplemented (Se-yeast 0.33 mg/kg) groups.

The results of baseline (week 2) vs. endpoint (week 8) whole-blood Se concentration in aging mice fed different concentrations of inorganic and organic Se are depicted in Figure S1 and Figure 2. Briefly, it was observed that the whole-blood Se concentration in the Se-D group was significantly (*p* < 0.05) decreased at week 8 compared to the week 2 baseline value (Figure S1) and the other groups (Figure 2). Similarly, at week 8, a small but statistically non-significant (*p* > 0.05) reduction in Se concentration was observed in the ISe-A and ISe-S groups compared to their week 2 baseline values (Figure S1). Whereas weeks 2 vs. 8 Se concentrations in OSe-A and OSe-S groups showed a stable trend, with relatively higher values in the latter group at week 8 (Figure S1). The comparative statistical analysis of endpoint (week 8) Se status further revealed that Se concentrations in the ISe-A, ISe-S, OSe-A, and OSe-S groups were significantly higher (*p* < 0.05) compared to the Se-D group. Meanwhile, no significant differences were observed in Se concentrations between ISe-S, OSe-A, and OSe-S groups at week 8 (Figure 2). However, as expected, Se concentration in OSe-S group was relatively higher than the ISe-A (*p* < 0.05), ISe-S, and OSe-A groups (*p* > 0.05) at week 8 (Figure 2).

### 3.3. Dietary Se Supplementation Improves Blood Total Antioxidant Capacity in Aging Mice

**Figure 3:**
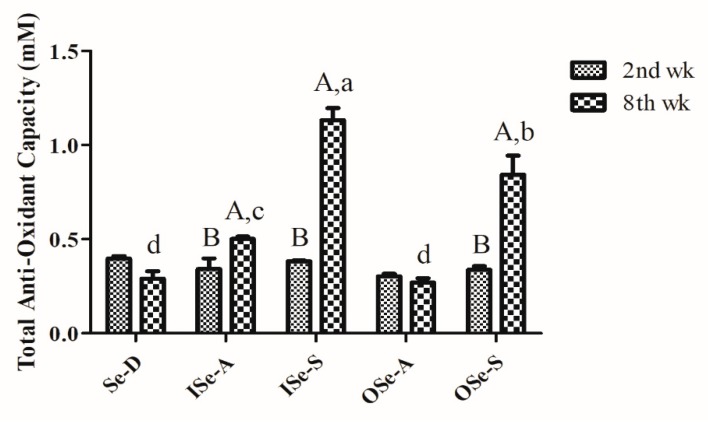
Baseline (week 2) and endpoint (week 8) blood total antioxidant capacity (TAOC) in aging female mice fed different concentrations of inorganic and organic selenium (Se). Values are expressed as mean ± SEMs. Different lowercase letters (a, b, c, and d) indicate significant difference (*p* < 0.05) between different groups, and different uppercase letters (A and B) indicate a significant difference (*p* < 0.05) between baseline (week 2) and endpoint (week 8) TAOC values within the same group. Each group comprised of three independent replicates. Notes: Se-D: Se-deficient (sodium selenite 0.08 mg/kg); ISe-A: inorganic Se-adequate (sodium selenite 0.15 mg/kg); ISe-S: inorganic Se-supplemented (sodium selenite 0.33 mg/kg); OSe-A: organic Se-adequate (Se-yeast 0.15 mg/kg); OSe-S: organic Se-supplemented (Se-yeast 0.33 mg/kg) groups.

As depicted in Figure 3, TAOC values at baseline (week 2) were comparable between all the groups and showed no significant differences. By week 8, TAOC in the Se-D group showed a relative decline compared to its baseline value; however, this difference was statistically non-significant. On the contrary, TAOC values in Se-supplemented groups (ISe-S and OSe-S) were significantly higher (*p* < 0.05) compared to the groups fed either a Se-D diet or ISe-A and OSe-A diets (Figure 3). Moreover, TAOC values were also significantly higher (*p* < 0.05) in the ISe-S group compared to the OSe-S group.

### 3.4. Dietary Se Supplementation Reduces Rate of Apoptosis in Ovarian Tissues of Aging Mice

**Figure 4:**
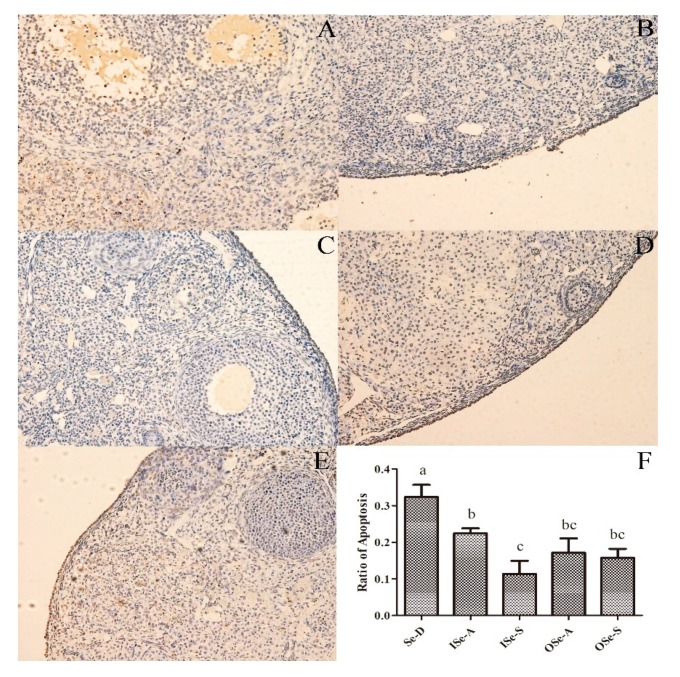
Apoptosis in ovarian tissues of aging female mice fed different concentrations of inorganic and organic selenium (Se). (**A–E**): Representative photomicrographs of ovarian sections showing TUNEL-positive cells in different groups. (**F**): Ratio of apoptosis (TUNEL-positive cells) in ovaries of aging female mice in different groups. Data are expressed as mean ± SEMs. Values with different lowercase letters (a, b, and c) indicate significant difference (*p* < 0.05). (**A**): Se-deficient (Se-D; sodium selenite 0.08 mg/kg); (**B**): inorganic Se-adequate (ISe-A; sodium selenite 0.15 mg/kg); (**C**): inorganic Se-supplemented (ISe-S; sodium selenite 0.33 mg/kg); (**D**): organic Se-adequate (OSe-A; Se-yeast 0.15 mg/kg); (**E**): organic Se-supplemented (OSe-S; Se-yeast 0.33 mg/kg) groups. Original magnification 200×. TUNEL: terminal dexynucleotidyl transferase (TdT)-mediated dUTP nick end labeling.

The representative photomicrographs of ovarian sections of aged female mice showing TUNEL-positive signals are depicted in Figure 4. Results of TUNEL assay revealed that the rate of apoptosis (TUNEL-positive cells) in ovarian tissues was significantly higher (*p* < 0.05) in the Se-D group compared to the Se-adequate (ISe-A and OSe-A) and Se-supplemented (ISe-S and OSe-S) groups (Figure 4). Notably, a relatively lower rate of apoptosis was observed in both Se-supplemented (ISe-S and OSe-S) groups (Figure 4). These findings indicate that Se deficiency can lead to an increased apoptosis rate, and that Se supplementation at an appropriate level can reasonably mitigate the occurrence of apoptosis in ovaries of aging female mice.

### 3.5. Effects of Dietary Se Supplementation on Maintenance of Follicle Reserves in Aging Ovaries

**Figure 5:**
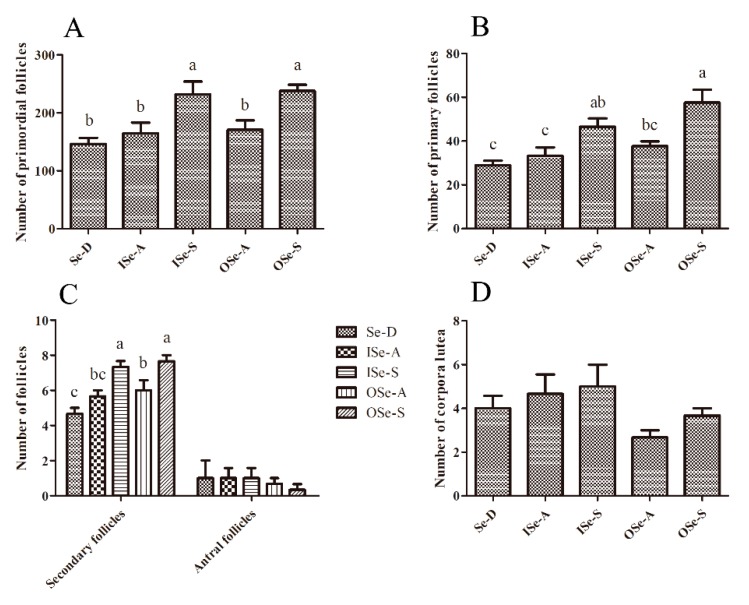
Follicle counts in ovaries of aging female mice fed different concentrations of inorganic and organic selenium (Se). (**A)**: Number of primordial follicles; (**B)**: Number of primary follicles; (**C**): Numbers of secondary and antral follicles; (**D**): Number of corpora lutea in different groups. Notes: Se-D: Se-deficient; ISe-A: inorganic Se-adequate; ISe-S: inorganic Se-supplemented; OSe-A: organic Se-adequate; OSe-S: organic Se-supplemented. Data are expressed as mean ± SEMs (*n* = 3 in each group). Bars with different letters (a, b, and c) indicate a significant difference (*p* < 0.05).

The representative photomicrographs of ovarian sections of aged female mice fed different concentrations of dietary Se are shown in Figure S2. As shown in Figure 5, our light microscopic stereological assessment of ovarian sections revealed that the numbers of primordial and primary follicles were relatively lower in the Se-D group compared to the Se-adequate (ISe-A and OSe-A; *p* > 0.05) and Se-supplemented (ISe-S and OSe-S; *p* < 0.05) groups. Although the differences in numbers of primordial and primary follicles between the Se-D and both Se adequate (ISe-A and OSe-A) groups were statistically non-significant, the latter groups showed slightly higher numbers of these follicles compared to the Se-D mice. This finding clearly shows that Se deficiency (or low levels) can contribute to an accelerated age-related depletion of primordial and primary follicles in females of an advancing age. However, as expected, significantly higher (*p* < 0.05) numbers of primordial and primary follicles were observed in both Se-supplemented (ISe-S and OSe-S) groups compared to the Se-D group. Meanwhile, the numbers of secondary (growing) follicles were also significantly higher (*p* < 0.05) in both Se-supplemented groups (ISe-S and OSe-S) compared to the Se adequate (ISe-A and OSe-A) and the Se-D groups. The numbers of antral follicles and corpora lutea showed a similar trend and no statistically significant differences were observed between all the groups (*p* > 0.05).

These findings reasonably indicate that low-Se (Se-D) levels can have a negative impact on the maintenance and potentially the development of ovarian follicles, and that both sources of dietary Se (inorganic and organic Se) exhibited comparable effects and can reasonably mitigate the age-related quantitative decline in otherwise compromised ovarian follicle reserves in 12–14 month old mice.

### 3.6. Effect of Dietary Se on Expression of Selenoprotein, Cell-Cycle, and Apoptosis-Related Genes in Ovaries of Aging Mice

**Figure 6:**
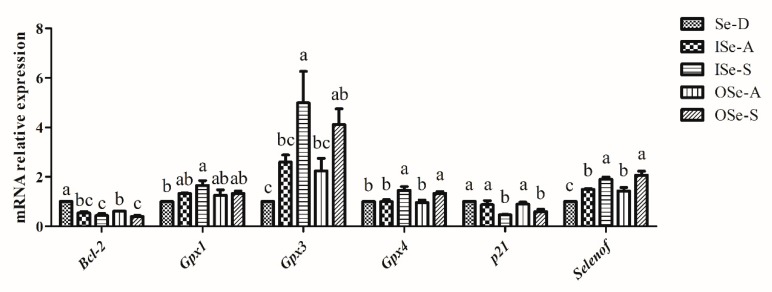
Relative mRNA expression levels of selenoprotein, cell-cycle-, and apoptosis-related genes in ovaries of aging mice fed different concentrations of inorganic and organic selenium (Se). Se-D: Se-deficient (sodium selenite 0.08 mg/kg); ISe-A: inorganic Se-adequate (sodium selenite 0.15 mg/kg); ISe-S: inorganic Se-supplemented (sodium selenite 0.33 mg/kg); OSe-A: organic Se-adequate (Se-yeast 0.15 mg/kg); OSe-S: organic Se-supplemented (Se-yeast 0.33 mg/kg) groups. Data are expressed as mean ± SEMs. Values with different lowercase letters (a, b, and c) indicate a significant difference (*p* < 0.05) between groups within the same variate (gene). *Gpx1*: glutathione peroxidase 1; *Gpx3*: glutathione peroxidase 3; *Gpx4*: glutathione peroxidase 4; *Selenof*: selenoprotein F; *Bcl-2*: B-cell leukemia/lymphoma 2; *p21* (also called *Cdkn1a*): cyclin-dependent kinase inhibitor 1A.

Briefly, our RT-qPCR results revealed that mRNA expression of selenoprotein (*Gpx1*, *Gpx3*, *Gpx4*, and *Selenof*), cell-cycle- (*p21*: also called “*Cdkn1a*”: cyclin-dependent kinase inhibitor 1A), and apoptosis-related (*Bcl-2*) genes in ovaries of aging mice was differentially modulated by dietary Se levels (Figure 6). Expression of *Gpx1* was relatively lower in the Se-D group compared to the Se-adequate (ISe-A and OSe-A) and Se-supplemented (ISe-S and OSe-S) groups. Notably, compared to the Se-D group, *Gpx1* expression was significantly upregulated (*p* < 0.05) in ISe-S group. In the Se-adequate (ISe-A and OSe-A) and Se-supplemented (OSe-S) groups, although its expression was upregulated compared to the Se-D group, no significant differences were observed between the groups (Figure 6). Similarly, the expression of *Gpx3* was significantly downregulated (*p* < 0.05) in the Se-D group compared to both ISe-S and OSe-S groups. Moreover, *Gpx3* expression levels were relatively higher in both Se-adequate (ISe-A and OSe-A) groups, but the difference was not statistically significant between the Se-D group and both Se-adequate groups (Figure 6). Interestingly, the expression of *Gpx4* remained stable in the Se-D and both Se-adequate (ISe-A and OSe-A) groups; however, its expression was significantly upregulated (*p* < 0.05) in the ISe-S and OSe-S groups, with a slightly higher expression levels observed in the ISe-S group (Figure 6). As for the expression of *Selenof*, compared to the Se-D group, significantly higher (*p* < 0.05) expression levels were observed in the Se-adequate (ISe-A and OSe-A) and Se-supplemented (ISe-S and OSe-S) groups. Specifically, significantly higher (*p* < 0.05) expression levels of *Selenof* were observed in both Se-supplemented groups (ISe-S and OSe-S) compared to the Se-D and both Se-adequate (ISe-A and OSe-A) groups (Figure 6).

Expression of *Bcl-2* was significantly lower (*p* < 0.05) in the Se-adequate (ISe-A and OSe-A) and Se-supplemented (ISe-S and OSe-S) groups compared to the Se-D group, in which a higher mRNA expression was observed (Figure 6). Intriguingly, the expression of *p21* was also differentially regulated following feeding with different Se concentrations. It was observed that its expression was significantly downregulated (*p* < 0.05) in both Se-supplemented (ISe-S and OSe-S) groups compared to the Se-D group and both Se-adequate (ISe-A and OSe-A) groups (Figure 6).

### 3.7. Expression of GPX4 protien in Ovarian Tissues of Aging Mice

**Figure 7:**
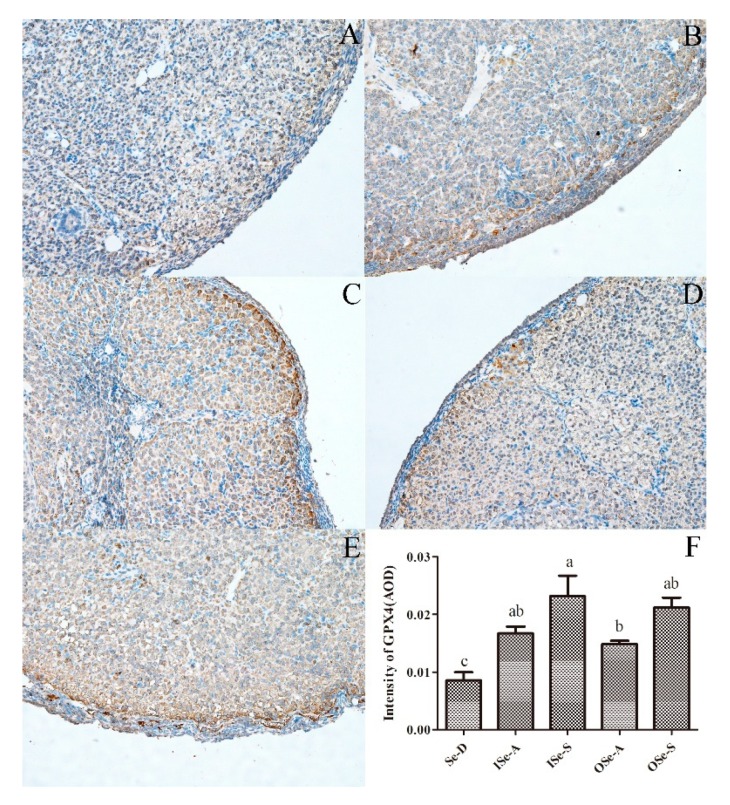
Expression of glutathione peroxidase 4 (GPX4) in ovarian tissue of aging mice fed different concentrations of inorganic and organic selenium (Se). (**A**–**E**): representative photomicrographs of ovarian sections (IHC) showing GPX4-positve signals in different groups. (**A**): Se-deficient (Se-D; sodium selenite 0.08 mg/kg) (**B**): inorganic Se-adequate (ISe-A; sodium selenite 0.15 mg/kg); (**C**): inorganic Se-supplemented (ISe-S; sodium selenite 0.33 mg/kg); (**D**): organic Se-adequate (OSe-A; Se-yeast 0.15 mg/kg); and (**E**): organic Se-supplemented (OSe-S; Se-yeast 0.33 mg/kg) groups. (**F**): Expression (pixel intensity) of GPX4 in ovarian sections of mice in different groups. AOD: average optical density. GPX4: glutathione peroxidase 4. Data are expressed as mean ± SEMs. Values with different lowercase letters (a, b, and c) indicate a significant difference (*p* < 0.05). Original magnification 200×.

In line with our RT-qPCR results, IHC analysis revealed that the expression signals of GPX4 protein in ovarian tissues were significantly higher (*p* < 0.05) in both the Se-adequate (ISe-A and OSe-A) and Se-supplemented (ISe-S and OSe-S) groups compared to the Se-D group (Figure 7). Interestingly, in coherence to its mRNA expression, GPX4 protein expression was also relatively higher in mice supplemented with sodium selenite 0.33 mg/kg (ISe-S) compared to those supplemented with Se-yeast 0.33 mg/kg (OSe-S), although this difference was statistically non-significant (Figure 7).

### 3.8. Effect of Se Supplementation on In Vitro Developmental Potential of Embryos Resulting from GV Oocytes

In the next experiment, we sought to evaluate the potential impact of dietary Se levels on in vitro development potential of embryos derived from GV oocytes in an aging mouse model. Detailed results along with the statistical significance between all the groups are shown in Table 1. Briefly, our results revealed that the IVM rates of GV oocytes showed small but statistically significant differences between the groups, with higher percentages of MII oocytes observed in OSe-A and OSe-S groups compared to the rest of the groups. The activation rate (PA) of MII oocytes was significantly higher in the Se-D group (100 ± 0%), and these values in the ISe-A, ISe-S, OSe-A, and OSe-S groups were comparable, where only the OSe-S group showed a small but statistically significant difference compared to the ISe-A group. The rates of two-cell embryos exhibited a similar trend and showed no significant differences between all the groups. Intriguingly, it was observed that despite a higher rate of two-cell embryos in the Se-D group, oocytes in this group showed a significantly diminished competence to develop to the blastocyst stage and beyond; this was further evident by the fact that none of the embryos survived to the hatched blastocyst stage in the Se-D group.

In contrast, Se supplementation (ISe-S and OSe-S) substantially improved the development potential of embryos resulting from in vitro matured GV oocytes compared to the Se-D group (*p* < 0.05). Notably, a significantly higher (*p* < 0.05) percentage of embryos developed to blastocyst and hatched blastocyst stages was observed in both Se-supplemented (ISe-S and OSe-S) groups. These results reasonably demonstrate that supplemental Se, regardless of its source, can ameliorate the in vitro developmental competence of embryos resulting from GV oocytes retrieved from aging mouse ovaries.

## 4. Discussion

Despite a considerable advancement in strategies intended for ameliorating the female reproductive efficiency and in vitro development of oocytes and embryos, there is a dearth of clinically viable strategies to either maintain or ameliorate the quantity and quality of oocytes during aging. It is important to note that among other aspects, age-related decline in both quality and quantity of oocytes is a critical rate-limiting factor with a strong bearing on the success rate of IVF and pregnancies. In the present study, using a mouse model of reproductive aging, we for the first time report that dietary Se supplementation can ameliorate female reproductive efficiency in aging females, potentially via modulation of expression of key antioxidant selenoprotein genes at the level of ovaries.

### 4.1. Blood Se Status and Total Antioxidant Capacity

In the current study, whole-blood was used to assess the Se status as it provides a longer-term estimate of Se status compared to plasma/serum [31,56]. As expected, a significantly lower whole-blood Se concentration was observed in Se-deficient mice compared to the Se-adequate and Se-supplemented groups at the study endpoint i.e., week 8. This finding is supported, to some extent, by a recent report [57] where it was shown that plasma Se concentration significantly declined in telomere-dysfunctional aged female mice (C57BL/6) fed a Se-deficient diet (Se-yeast 0.03 mg Se/kg) compared to those fed an adequate inorganic-Se diet (0.15 mg Se/kg) until aged 18 or 24 months; this decline was more pronounced in the latter age group. Similarly, in two very recent studies [42,43] on young female BALB/c mice, a significant reduction in whole-blood Se levels was reported following a three-month long feeding with a Se-deficient (0.02 mg Se/kg) diet. On the contrary, in both studies, mice fed Se-adequate (0.15 mg Se/kg) and high-Se (1.5 mg Se/kg) diets showed significantly higher levels of whole-blood concentrations, however, both studies did not report baseline Se values [42,43]. Interestingly, in the present study, compared to baseline Se values, a small fractional decline in whole-blood Se levels was observed in mice fed inorganic form of Se in Se-adequate and Se-supplemented groups. However, mice fed an organic form of Se in both adequate and supplementation groups showed a stable whole-blood Se level compared to their respective baseline values. There is a dearth of scientific evidence, in particular from aging models, with which to compare our whole-blood Se levels as observed in the Se-adequate and Se-supplemented groups fed organic and inorganic sources. However, it is believed that once the optimal selenoprotein biosynthesis is achieved further increase in Se intake is offset almost completely by excretion via urine, feces, and breath, and only minimal increase in whole-body Se levels could be achieved. It is also worthwhile to mention that specific/regulated Se pool (selenoproteins and a small-molecule metabolites) is sharply circumscribed and further expansion by means of tissue deposition is unlikely with increasing Se intake (reviewed in [12]). In coherence with this notion, our findings regarding whole-blood levels in Se-adequate and Se-supplemented aged mice could infer that Se status and selenoprotein biosynthesis might have achieved a plateau in these groups, and thus excess Se was possibly excreted via a whole-body Se regulation mechanism as outlined above and greatly discussed elsewhere [12].

Blood TAOC is considered as a useful marker for assessing the antioxidant function, reflecting the capacity to compensate exogenous and endogenous (free radical) stresses [58]. A previous study has shown that aging is implicated in impairing the TAOC in different brain tissues in rats [59]. In the current study, we observed that blood TAOC was significantly higher in Se-supplemented mice than the Se-deficient and Se-adequate groups. Although there is a comparative dearth of evidence regarding Se-effect on blood TAOC in aging models, our results are partly supported by findings of a recent study [60] on aging mice, where it was shown that high-Se rice feeding significantly enhanced the liver TAOC levels in aging mice. In addition, 12-week Se supplementation significantly increased plasma TAOC levels in pregnant Iranian women at a risk for intrauterine growth restriction [33]. In a recent study [61], an improved blood TAOC was observed in Iranian women with polycystic ovary syndrome following 12-week Se supplementation. In any case, in coherence to our findings, several recent reports have also shown that Se addition (different sources) in diet can significantly enhance the blood TAOC in different non-aging animal models [58,62,63,64,65,66].

### 4.2. Effect of Se on Follicle Quantity and Apoptosis in Ovarian Tissue of Aging Mice

Folliculogenesis is considered as a critical developmental window for determining the quality of developing oocytes, because this is the prime phase during which female gametes acquire essential products from the maternal pool for driving their subsequent maturation, fertilization and early embryonic development. Therefore, it is likely that any perturbation occurring during follicular growth phase would undoubtedly affect the quality and competence of resulting oocytes [3]. Previous studies on aging mouse models have demonstrated that age-related accumulating oxidative stress and impaired antioxidant status and redox homeostasis are critical contributors to an increasing decline in both quantity and quality of follicles/oocytes [4,6,7]. In the present study, dietary Se-deficiency showed a negative impact on the stockpile of primordial follicles and numbers of primary and secondary follicles. Meanwhile, Se supplementation considerably mitigated the age-related decline in follicle reserves. It is important to note that our understanding of the precise molecular basis of these observations is limited, largely due to the fact that there is very little evidence available on potential implications of Se and selenoproteins in mammalian ovarian function (reviewed in [9]). However, considering the canonical antioxidant role of Se and several selenoproteins [10], it is reasonable to infer that the ameliorative effects of Se regarding follicle reserves as observed in the current could be attributed to mitigation of age-related oxidative stress and apoptosis and/or other types of cell death at the level of ovaries. At the same time, these ameliorative effects with regard to the improved follicle numbers can also be attributed to the potential role of Se and selenoproteins in proliferation of granulosa cells [9].

The growth and development of an oocyte is a highly coordinated and complex biological process and largely dependent on surrounding granulosa and stromal cells. Interestingly, follicles isolated from aging mice ovaries show perturbations in gene expression and hormonal profiles and contain oocytes with diminished meiotic competence which is highlighted by a relatively higher incidence of spindle defects compared to their younger counterparts [67]. Indeed, the growth of granulosa cells has been considered as a crucial indicator of follicle development process. It was shown that Se supplementation can modulate the proliferation of bovine granulosa cells in vitro, potentially via stimulating estradiol synthesis and repressing nitric acid production [68]. Similarly, Se supplementation was also reported to stimulate the proliferation of bovine luteal cells, and these effects were attributed to Se-mediated amelioration of steroid hormones-related lipid peroxidation [69]. Until recently, precise molecular basis of Se-mediated effects on granulosa and theca cells reported in two previous studies [68,69] remain largely unclear. Moreover, recent advancement in the ovarian research has improved our understanding of different intra-ovarian molecular mechanisms implicated in maintaining the healthy quiescence and activation of the primordial follicle reserve. PI3K (phosphatidylinositide 3-kinases) pathway has been implicated to play an important role in integration of several known and unknown factors principally associated with maintenance of primordial follicle dynamics [70]. Interestingly, in a recent in vitro cell culture study, it was shown that Se supplementation can stimulate the proliferation of caprine luteinized-granulosa cells and steroidogenesis potentially via modulating PI3K/Akt and AMP-activated protein kinase (AMPK) pathways and expression of downstream genes related to cell cycle and steroidogenesis, and improving the cellular oxidative status [45]. In addition, a recent pilot study [28] on bovine ovaries has shown that Se was consistently localized in the granulosa and theca cells of healthy and atretic follicles. Further quantification resolved that concertation of Se was significantly higher in healthy follicles than in the corpora lutea (26 ± 5.0 vs. 2.6 ± 1.3 ppm) [28]. It was also suggested that Se, invariably in the form of antioxidant selenoproteins, could combat the oxidative- and endoplasmic reticulum (ER) -related stresses during follicular development and consequently mitigate the potential DNA damage and atresia in oocytes [28]. It is well-recognized that large follicles express a higher quantity of cytochrome P450s, and that these enzymes are required for steroid hormone (P_4_ and estradiol) synthesis in dominant follicles and eventually produce free radicals and H_2_O_2_, contributing to an accumulating oxidative burden within ovaries [28]. It is also believed that selenoproteins such as GPX1, GPX3, GPX4, and SELENOS can scavenge the accumulating ROS and mitigate the oxidative stress during the follicle development [28]. However, the precise role of Se and selenoproteins in ameliorating the age-related decline in follicle reserves and perhaps the quality warrants further focused studies. It is worth mentioning that previous studies using mouse models of aging have also shown that age-related decline in follicle reserves could be, at least in part, ameliorated using antioxidant interventions, potentially via mitigating the oxidative stress [4,6,8]. Given that the levels of anti-Mullerian hormone (AMH), an important biomarker of ovarian reserves, are dramatically reduced during aging (12–14 months) and can mirror the age-related decile in primordial follicle reserves [71], it would be interesting to see if the future studies are complemented by evaluation of AMH concentration at the level of ovaries.

Furthermore, as for the role of Se in mitigating apoptosis, it has been reported that Se can reduce oxidative stress-related apoptotic cell death, potentially via antioxidant and antiapoptotic roles of several selenoproteins (reviewed in [9,10]). Indeed, our current findings also revealed that dietary Se-deficiency resulted in a significantly higher ratio of TUNEL-positive cells in the ovarian tissues of aging mice, whereas Se-supplementation substantially reduced this ratio. Recently, it was shown that Se can mitigate oxidative stress-related apoptosis in brain tissue of aging female rats, potentially by improving the GPX activity [72]. Similar findings were observed in another recent study where it was demonstrated that Se can reduce aflatoxin-induced apoptosis rate by modulating the death receptor- and ER pathways-related molecules in chicken jejunal cells [73]. Interestingly, Se deficiency also led to perturbations in redox signaling and activation of NF-κB signaling, resulting in apoptosis, via intrinsic and extrinsic pathways, in chicken duodenal villi cells [74]. Previous cell culture studies have also demonstrated that Se supplementation can significantly reduce the rate of apoptosis in placental trophoblast cell lines: BeWo, JEG-3, and Swan-71 [75]. In addition, a significant reduction in apoptosis rate was observed when porcine parthenotes were in vitro produced in culture media supplemented with inorganic Se [76].

### 4.3. Se Effect on In Vitro Embryo Developmental Potential

Although the precise mechanistic basis behind age-related decline in oocyte quality is poorly understood, it is a well-recognized fact that oxidative stress is a main contributor to this phenomenon [77]. It is also well-established that oocytes with declined quality are likely to have a significantly reduced competence for early embryonic development. This decline in quality is largely related to the age-related increase in occurrence of meiotic defects and is also likely to be compounded by additional cytoplasmic perturbations in key physiologic states in oocytes such as mitochondrial function and protein homeostasis [3], and possibly in the lipid profiles in peri-ovarian microenvironment [67]. Nevertheless, it is now well understandable that the decline in oocyte quality and competence is multifactorial and a wholistic and generalized perspective covering all developmental stages of oocytes is needed to fully elucidate the underlaying aging mechanisms [3]. It is also argued that antioxidant intervention aimed at alleviating the oxidative insult in oocytes should be adopted in vivo to mitigate the cumulative age-associated oxidative stress rather than as a late-stage therapy to alleviate the cellular perturbations in oocytes once such stressful manifestations have been established [77].

Intriguingly in the current study, we observed that the in vitro developmental potential of GV oocytes-derived embryos (blastocysts and hatched blastocysts %) was significantly affected in the Se-D aged mice. Percentages of blastocysts and hatched blastocysts were significantly improved when mice were supplemented with both sources of Se (see Table 1). Indeed, the loss of oocyte quality and its subsequent developmental potential has a correlative link to the overall female fertility, as it was shown previously that the rate of live birth per oocyte was significantly reduced (1% vs. 26%) in aging women (aged ≥ 42 years) compared to their younger counterparts [78]. It is generally well-established that Se deficiency impacts the level of selenoprotein synthesis [79]. It was shown that the inability of homozygous *Trsp* (–/–) embryos to synthesize selenoproteins resulted in their early embryonic death in germ-line mutant mice [80]. In addition, it has been suggested that Se availability itself is an important factor for determining the extent of selenoprotein synthesis in embryos; therefore it is necessary that Se should be maternally supplied to the embryos for adequate selenoprotein synthesis [81]. It has also been argued that Se content of pre-ovulatory oocytes is likely to be affected by the systemic impact of maternal blood Se levels, and that the pre-ovulatory (maternal) Se content is potentially a main source of Se for selenoprotein synthesis in oocytes and pre-implantation embryos [82]. To some extent, this is supported by the fact that oocytes grow in follicular fluid which itself is a product of blood-derived constituents, and that the follicular microenvironment also plays an essential role in folliculogenesis and fertility [29], as it was previously shown that patients with idiopathic infertility had significantly reduced follicular Se levels [29].

It has also been suggested that regardless of the source of maternal dietary Se, Se content in embryos would come from an organic metabolite: either from SeMet pool of oocytes and/or oocyte selenoproteins [47]. Therefore, it can be hypothesized that oocyte (maternal) selenoproteins provide a common source of SeCys for embryo selenoprotein synthesis [47]. Interestingly, this hypothesis is partly supported by the findings of two recent studies, where higher Se level and GPX activities were observed in embryos harvested from Se-supplemented gilts, reinforcing the notion that embryos, regardless of maternal dietary Se source, may obtain their Se content from a common intermediary Se metabolite [83,84]. In this way, although the fundamental basis still remains unclear, it could be inferred that more Se might be available to oocytes and embryos resulting from Se-supplemented aged mice in the current study. In addition, the improved developmental potential of oocytes and embryos in Se-supplemented aged mice may also be attributed to an improved antioxidant status in ovaries, oocytes, and their resulting embryos. This notion is also partly supported by the fact that the canonical antioxidant role of Se is performed by selenoproteins [9,10]. Interestingly, Se-levels differentially modulated the mRNA expression of antioxidant selenoproteins (discussed later) in mice ovaries in this study, potentially contributing to an improved antioxidant status. These arguments are also supported by the recent findings of Ceko et al. [28] who reported that a significantly higher *GPX1* expression was observed in cumulus cells recovered from human oocytes yielding a successful pregnancy (*n* = 12) in an IVF/intracytoplasmic sperm injection (ICSI) program upon embryo transfer. In an in vitro study [44], significantly improved blastocyst rate was observed when yak oocytes were supplemented with Se (2 and 4 μg/mL) during IVM. Similarly, in another in vitro culture study [76] on porcine parthenotes, a higher rate of blastocysts, cell number, and inner cell mass (ICM) rate were observed when Se (25 and 250 ng/mL sodium selenite) was added to the culture media. Se supplementation (10 ng/mL sodium selenite) in culture media was also able to improve the in vitro maturation rate of mouse preantral follicles [85]. Same group also reported an increased in vitro developmental potential of vitrified mouse preantral follicles following Se supplementation in in vitro culture media [86]. At the same time, pre-gestational and gestational maternal Se supplementation (3.0 μg/day) significantly improved the blastocyst rate and pre-implantation success in ICR mice [50]. Although the literature on the effect of Se on improving the in vitro development potential of oocytes and embryos in aging females is lacking, researchers have shown that other antioxidants such as resveratrol, MT, and NAC can significantly ameliorate the oocyte quality and embryo development in aging mouse models [4,6,8].

### 4.4. Expression of Gpx1, Gpx3, Gpx4, Selenof, Bcl-2, and p21

Dietary Se deficiency and oxidative stress have been linked to selective regulation of several selenoproteins involved in antioxidant defense and redox homeostasis [87,88], and such regulation is believed to be tissue-specific [12,89]. Indeed, in the current study, the expression of *Gpx1*, *Gpx3*, and *Gpx4* was differentially regulated in ovaries of aging mice. Relatively lower and higher expression of these selenoproteins was detected in Se-D and Se-supplemented groups, respectively. Similarly, recently it was shown that Se deficiency downregulated several selenoproteins, including *Gpx1*, *Gpx3*, and *Gpx4*, in an organ-specific manner in mice [89]. Interestingly, long-term Se deficiency and age also significantly downregulated mRNA levels of many selenoproteins such as *Gpx1*, *Gpx3*, and *Gpx4* in a tissue-specific manner in aged female G3 *Terc* ^−/−^ C57BL/6 mice [57]. In any case, precise fundamental basis of this tissue-specific hierarchical selenotranscriptomic regulation by dietary Se deficiency still remains poorly elucidated [57]. In general, GPX1, GPX3, and GPX4 are well-recognized for their antioxidant and redox regulatory roles, and have been implicated in playing significant roles in mammalian fertility and reproduction [9,10]. Expression of *GPX1*, *GPX3*, and *GPX4* was observed in small and large healthy and atretic bovine follicles and corpora lutea and it was suggested that these selenoproteins, in particular *GPX1*, play important roles in follicle growth and dominance, potentially via their canonical antioxidant function [28]. Similarly, COCs from oocyte yielding successful pregnancy in women also showed higher expression of *GPX1* [28]. In addition, granulosa cells in small atretic bovine follicles had a lower expression of *GPX3* compared to the healthy follicles [90]. In fact, *Gpx3* showed a positive correlation with progesterone and estrogen and was highly expressed in mouse decidua (days 5–8 of pregnancy) [91]. It has also been implicated in postovulatory process of endometrial remodeling, potentially via mitigating oxidative stress [91]. *GPX3* was suggested to play a role in mitigating oxidative stress-related apoptosis in growing healthy follicles in Japanese Black cows [92]. This evidence, together with the findings of the current study, reasonably reinforce the notion that these selenoproteins have essential roles in female fertility and reproduction, which need to be precisely elucidated at individual levels.

Se regulation of *Gpx4* in the current was particularly intriguing, as both mRNA and protein expressions (IHC) of GPX4 were significantly higher in Se-supplemented group than the Se-D group. Although GPX4 has a well-recognized role in male fertility (reviewed in [10]), its precise implication in female reproduction is poorly elucidated, and only a few studies have shown its relevance to human pregnancy-related complications (reviewed in [9]). Intriguingly, as a lipid peroxidase, GPX4 is the only peroxidase that reduces toxic lipid peroxides to non-toxic lipid alcohols in animal cells, and this task is achieved with the critical involvement of two glutathione (GSH) molecules [93]. This process prevents the iron-dependent formation of toxic lipid-ROS, and the functional inactivation of GPX4 results in an increased lipid-ROS accumulation and subsequent lipid peroxidation [94]. Indeed, current insights into the role of GPX4 has further improved our understanding of this selenoprotein in tissue redox homeostasis [95,96,97,98,99]. Together with its role in lipid peroxidation, GPX4 is also considered as an essential regulator of physiologically important signaling lipids, and a balance between both of these functions is critical in maintaining homeostasis within a given cell (reviewed in [94]). To this effect, recent research has indeed shown that GPX4 is implicated in regulating ferroptosis (a form of necrotic cell death) in different epithelial cells lines, potentially by enzymatic scavenging of hydroperoxy lipids [100]. Inactivation of GPX4 has been linked to phospholipid peroxidation-mediated cell death, and due its ability to mitigate phospholipid peroxidation and ferroptosis, GPX4 has been regarded as an indispensable selenoproteins and cannot be replaced by any of the other redox-active enzymes. Thus, it is considered as a limiting selenoprotein for mammalian cell proliferation and survival, at least at the level of cell culture [96].

In addition to *Gpxs* 1, 3 and 4, our results also revealed a relatively lower expression of *Selenof* in response to a low-Se diet, whereas its expression was significantly higher in both Se-adequate and Se-supplemented groups. As a “thioredoxin-like” selenoprotein, SELENOF is believed to perform oxidoreductase roles and is differentially expressed under adaptive and acute cellular stress conditions and changes to the Se levels [101,102,103]. It has been shown that, as an ER-resident selenoprotein, SELENOF is implicated in unfolded protein response (UPR), protecting the accumulation of unfolded or misfolded proteins in ER and thereby mitigating ER stress in cells [101]. Similarly, persistent ER stress could also lead to cellular apoptosis, therefore, SELENOF may also play its role in reducing oxidative- and ER-related stress. Indeed, *SELENOF* has been implicated in mitigating tunicamycin-induced apoptosis in human lens epithelial (hLE) cells, potentially via inhibiting mitochondria-dependent oxidative stress [104]. In a recent selenotranscriptomic study, it was shown that long-term Se deficiency and age downregulated the mRNA expression of *Selenof* in a sex- and tissue-specific pattern in telomere-dysfunctional mouse model [57]. Moreover, an upregulated mRNA expression of *SELENOF* was reported in growing lambs supplemented with inorganic Se [105]. In any case, these findings reasonably highlight that expression of *Selenof* is regulated in a tissue-specific manner and responds differentially to Se levels. Our understanding of the regulation of this selenoprotein, both at transcriptional and translational levels, in different stress-related and pathologic conditions [102,103] is insufficient and further focused studies will help understand its physiological function in detail.

In the current study, an interesting phenomenon regarding mRNA expression of *Bcl-2* was observed. In the Se-D group, an overexpression of *Bcl-2* was observed compared to the Se-adequate and Se-supplemented groups. In general, the Bcl-2 family has been implicated in regulating the complex interactions of several anti-apoptotic and pro-apoptotic molecules, governing the apoptosis-activation threshold at cellular levels [106,107]. On the contrary, previous reports have shown that Bcl-2 can also modulate the process of autophagy [107,108]. Although autophagy is generally considered as a pro-survival mechanism [109], at non-physiological levels and in response to different cellular stresses it can lead to cell death [107]. For instance, it has been suggested that autophagy is drastically induced in response to nutrient-limiting conditions [107], and its regulation is also mediated by various patho-physiological events and signals [110]. It has been shown that Bcl-2 can inhibit the autophagy via its strong independent interaction with Beclin 1 in a highly regulated manner in yeast and mammalian cell lines [107]. Similarly, in a recent report [108], it was demonstrated that Bcl-2, by inhibiting Beclin 1, could result in the release of pro-apoptotic proteins i.e., Bak and Bax to promote apoptosis via activation of effector caspase-3 in atretic follicles in rat ovaries. It was also speculated that this interaction between Beclin 1 and Bcl-2 (or perhaps other Bcl-2 family members) could impair the ability of Bcl-2 to inhibit the pro-apoptotic proteins i.e., Bax and Bak, which in turn, might trigger cell apoptosis [108]. Recently it was also shown that Se deficiency led to an increased mRNA and protein expression of Bcl-2 and inhibited autophagy and induced apoptosis in chicken cardiomyocytes by inhibiting Bax/Bcl-2 and caspases-mediated cleavage of Beclin 1 [111]. Similarly, an increased mRNA and protein expression of Bcl-2 was observed in *SELENOU* knocked-down chicken Sertoli cells [112]. This evidence indicates that, due to its pleotropic nature, Bcl-2 plays many regulatory roles (canonical and non-canonical) depending on cell type and stimuli [107,112]. Besides, we observed that mRNA expression of cell cycle-related *p21* gene was significantly modulated following Se-supplementation. As an inhibitor of cyclin-dependent kinases (CDKs), p21 has been shown to impair G1-to-S-phase cell cycle progression via modulating cell cycle check-points [113]. In a recent in vitro study [45], it was shown that Se supplementation (5 ng/mL) improved proliferative capacity of luteinized granulosa cells, potentially via downregulation of P21 expression both at mRNA and protein levels. Similarly, Se-deficient and Se-excess diets also resulted in cell cycle arrest during spermatogenesis in mice, potentially via elevated expression of p21 and decreased expression of CDC_2_ and Cyclin B1 [113].

## 5. Conclusions

In the current study we demonstrate that Se deficiency negatively impacted the whole-blood Se status and follicle development, and increased the rate of apoptosis in ovaries of aged mice. Conversely, these biomarkers were substantially ameliorated in Se-supplemented (inorganic and organic) groups. At the same time, mRNA expression of *Gpx1*, *Gpx3*, *Gpx4* (also its protein expression)*,* and *Selenof*, *p21,* and *Bcl-2* genes in ovaries of aging mice was differentially modulated by dietary Se levels. Intriguingly, the developmental potential of embryos resulting from GV oocytes retrieved from ovaries of Se-D mice was also significantly reduced compared to the Se-supplemented groups in which significantly higher blastocyst and hatched blastocyst percentages were observed. Collectively, our findings indicate that Se supplementation positively modulated the antioxidant status and reduced the rate of apoptosis in aging mice, thereby improving the in vitro developmental potential of embryos resulting from GV oocytes, potentially via modulation of expression of *Gpx1*, *Gpx3*, *Gpx4, Selenof, p21,* and *Bcl-2* genes at the level of the ovaries. Based on these results, it is envisaged that Se deficiency can negatively impact ovarian function, and that Se supplementation at an appropriate level can substantiate ovarian function and female fertility, particularly in aging subjects.

Indeed, our results add more value to the tally of the already known benefits of this trace mineral (Se) and will pave a way for further mechanistic and larger and viable intervention studies focused on ameliorating ovarian function and overall female fertility and reproduction in the future. However, considering the U-shaped response curve of Se, one should be very careful in determining the optimal level of Se for such studies.

## Figures and Tables

**Table 1:** In vitro developmental potential of embryos resulting from GV oocytes retrieved from ovaries of aging mice fed different concentrations of inorganic and organic selenium (Se).

Groups	Number of GV Oocytes Collected	M II Oocytes (%)	Activated Oocytes (%)	No. of Embryos Developed to		
				Two-Cell Embryos (%)	Blastocysts (%)	Hatched Blastocysts (%)
Se-D	43	26 (60.51 ± 0.89) ^bc^	26 (100 ± 0) ^a^	17 (63.89 ± 12.73)	5 (19.44 ± 4.81) ^b^	0 (0)^d^
ISe-A	33	17 (51.52 ± 2.62) ^d^	11 (82.22 ± 1.92) ^c^	9 (65.00 ± 8.66)	6 (36.67 ± 15.28) ^ab^	2 (13.33 ± 11.55) ^c^
ISe-S	59	34 (57.49 ± 1.10) ^cd^	28 (83.09 ± 5.28) ^bc^	18 (63.13 ± 11.77)	12 (42.47 ± 4.33) ^a^	10 (35.73 ± 2.15) ^a^
OSe-A	66	46 (69.70 ± 6.94) ^a^	40 (87.29 ± 5.28) ^bc^	27 (67.40 ± 5.19)	11(29.85 ± 6.37) ^ab^	7 (17.4 ± 3.49) ^bc^
OSe-S	58	38 (65.56 ± 5.09) ^ab^	34 (89.68 ± 3.43) ^b^	20 (58.59 ± 7.00)	13 (37.88 ± 11.44) ^a^	9 (26.52 ± 1.31) ^ab^

Notes: Se-D: Se-deficient (sodium selenite 0.08 mg/kg); ISe-A: inorganic Se-adequate (sodium selenite 0.15 mg/kg); ISe-S: inorganic Se-supplemented (sodium selenite 0.33 mg/kg); OSe-A: organic Se-adequate (OSe-A; Se-yeast 0.15 mg/kg); OSe-S: organic Se-supplemented (Se-yeast 0.33 mg/kg) groups. GV: germinal vesicle; M II: metaphase II. Values with different superscripts (a–d) within the same column are significantly different (*p* < 0.05). *n* = 6 mice in each group.

## Supplementary Materials

The following are available online at https://www.mdpi.com/2076-3921/8/12/634/s1. Figure S1: Baseline (week 2) vs. endpoint (week 8) whole-blood Se levels in aging female mice fed different concentrations of inorganic and organic selenium (Se). Figure S2: The representative photomicrographs of H and E stained ovarian sections of aged female mice fed different concentrations of dietary selenium (Se). Table S1: Details of primers used in RT-qPCR assay in this study.
