# Supplementary material for: Dietary Selenium Supplementation Ameliorates Female Reproductive Efficiency in Aging Mice
Source: Antioxidants (Basel). 2019 Dec 11;8(12):634. doi: 10.3390/antiox8120634 (PMC6969897; doi:10.3390/antiox8120634)
Supplement: Supplementary file 1 [file antioxidants-08-00634-s001.pdf]

antioxidants

MDPI

Article

Dietary Selenium Supplementation Ameliorates Female Reproductive Efficiency in Aging Mice

Haoxuan Yang [1,†], Izhar Hyder Qazi [1,2,†], Bo Pan [1], Christiana Angel [3,4], Shichao Guo[1], Jingyu Yang[1], Yan Zhang[1], Zhang Ming [1], Changjun Zeng [1], Qingyong Meng[5], Hongbing Han [6,*], and Guangbin Zhou [1,*]

[1] Farm Animal Genetic Resources Exploration and Innovation Key Laboratory of Sichuan Province, College of Animal Science and Technology, Sichuan Agricultural University, Chengdu 611130, China; yanghaoxuan940712@gmail.com (H.Y.); vetdr_izhar@yahoo.com (I.H.Q.); bopan1992@163.com (B.P.); daxiaopang@outlook.com (S.G.); yangjinyu19960710@163.com (J.Y.); yanzhang@sicau.edu.cn (Y.Z.); zhangming@sicau.edu.cn (Z.M.); zengchj@sicau.edu.cn (C.Z.)

[2] Department of Veterinary Anatomy and Histology, Shaheed Benazir Bhutto University of Veterinary and Animal Sciences, Sakrand 67210, Sindh, Pakistan

[3] Department of Veterinary Parasitology, College of Veterinary Medicine, Sichuan Agricultural University, Chengdu 611130, China; qazi5502@yahoo.com

[4] Department of Veterinary Parasitology, Faculty of Veterinary Sciences, Shaheed Benazir Bhutto University of Veterinary and Animal Sciences, Sakrand 67210, Sindh, Pakistan

[5] State Key Laboratory of AgroBiotechnology, China Agricultural University, Beijing 100193, China; qymeng@cau.edu.cn

[6] National Engineering Laboratory for Animal Breeding, Key Laboratory of Animal Genetics and Breeding of the Ministry of Agriculture, Beijing Key Laboratory for Animal Genetic Improvement, College of Animal Science and Technology, China Agricultural University, Beijing 100193, China

* Correspondence: hanhongbing@cau.edu.cn (H.H.); zguangbin@sicau.edu.cn (G.Z.); Tel.: +86-10-6273-2681 (H.H.); +86-159-081-89189 (G.Z.)

† Joint first authors with equal contribution to this work.

1. Supplementary material

Table S1. Details of primers used in RT-qPCR assay in this study.

Genes	Primer sequences (5'→3')	Tm (°C)	Product length (bp)	Accession ID
Gpx1	F: CACAGTCCACCGTGTATGCCTTC<br>R: ACCGAGCACCACCAGTCCAC	60	191	NM_008160.6
Gpx3	F: CGAGTATGGAGCCCTCACCA<br>R: GCCCAGAATGACCAAGCCAA	60	172	NM_008161.4
Gpx4	F: ATAAGACGGCTGCGTGGTGAAG<br>R: TAGAGATAGCACGGCAGGTCCTTC	60	81	NM_008162.4
Selenof	F: TGGACGACAACGGGAACATTGC<br>R: GCGTTCCAACTTCTCGCTCAGG	60	92	NM_053102.2
p21	F: TCCTGGTGATGTCCGACCTGTTC<br>R: CGGCGCAACTGCTCACTGTC	60	84	NM_001111099.2
Bcl-2	F: TCCTTCCAGCCTGAGAGCAACC<br>R: CCGGCGGAGGGTCAGATGG	60	141	NM_009741.5
Gapdh	F: CATGGCCTTCCGTGTTCCTA<br>R: GCCTGCTTACCACCTTCTT	60	104	NM_008084.3

Abbreviations/aliases: F: forward; R: Reverse; *Gpx1*: glutathione peroxidase 1; *Gpx3*: glutathione peroxidase 3; *Gpx4*: glutathione peroxidase 4; *Selenof*: Selenoprotein F; *Bcl-2*: B cell leukemia/lymphoma 2; *p21* also called *Cdkn1a*: cyclin-dependent kinase inhibitor 1A; *Gapdh*: glyceraldehyde 3-phosphate dehydrogenase; ID: identifier.

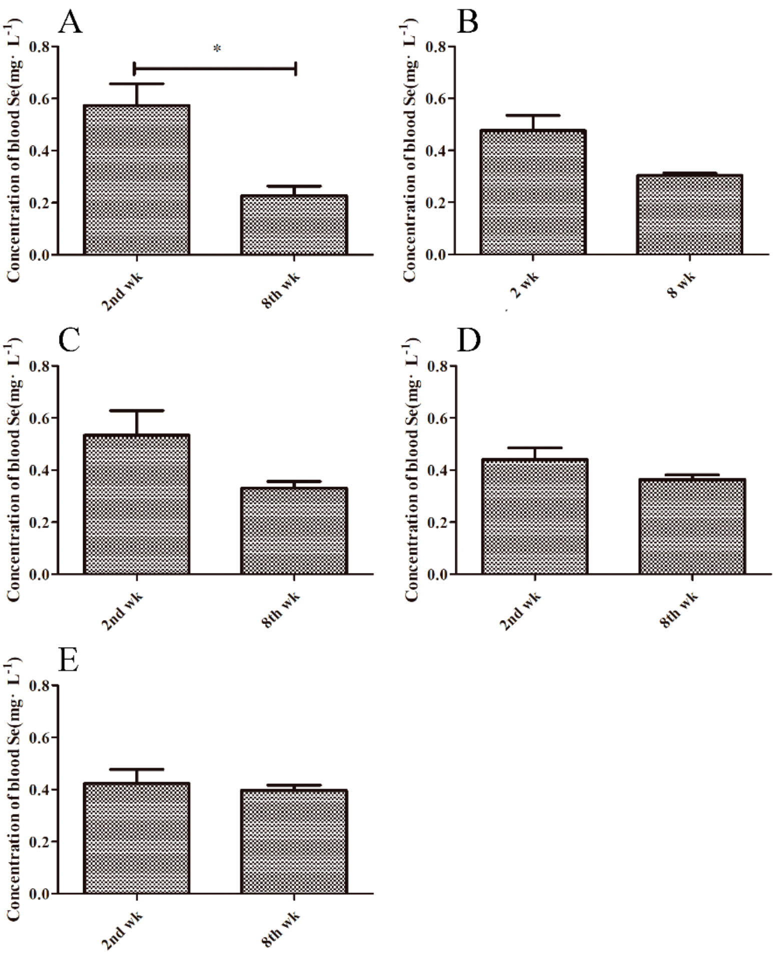

Figure S1. Baseline (week 2) vs. endpoint (week 8) whole-blood Se levels in aging female mice fed different concentrations of inorganic and organic selenium (Se). Values are expressed as mean ± SEMs. Each group comprised of three independent replicates. Bars with asterisk (*) indicates significant difference ($p < 0.05$) between baseline vs. endpoint whole-blood Se concentration within the same group. Notes: **(A)** Se-D: Se-deficient (sodium selenite 0.08 mg/kg); **(B)** ISe-A; inorganic Se-adequate (sodium selenite 0.15 mg/kg); **(C)** ISe-S: inorganic Se-supplemented (sodium selenite 0.33 mg/kg); **(D)** OSe-A: organic Se-adequate (Se-yeast 0.15 mg/kg); **(E)** OSe-S: organic Se-supplemented (Se-yeast 0.33 mg/kg) groups.

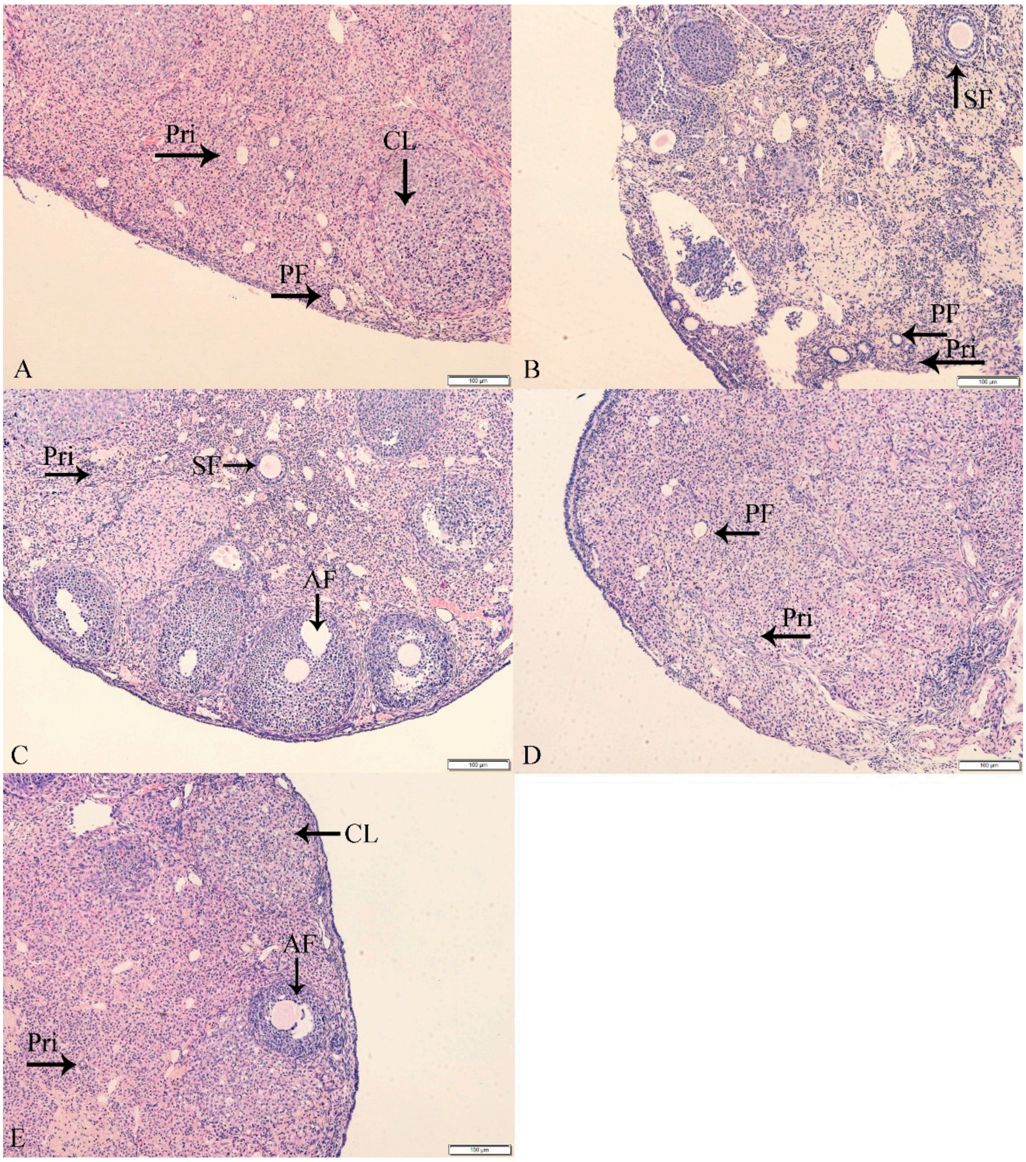

Figure S2. The representative photomicrographs of H and E stained ovarian sections of aged female mice fed different concentrations of dietary selenium (Se). **A**: Se-deficient (Se-D; sodium selenite 0.08 mg/kg); **B**: inorganic Se-adequate (ISe-A; sodium selenite 0.15 mg/kg); **C**: inorganic Se-supplemented (ISe-S; sodium selenite 0.33 mg/kg); **D**: organic Se-adequate (OSe-A; Se-yeast 0.15 mg/kg); **E:** organic Se-supplemented (OSe-S; Se-yeast 0.33 mg/kg) groups. **Notes**: Pri: primordial follicle; PF: primary follicle; SF: secondary follicle; AF: antral follicle; CL: corpus luteum. Original magnification 100 ×.
